# Brain Atrophy and Clinical Characterization of Adults With Mild Cognitive Impairment and Different Cerebrospinal Fluid Biomarker Profiles According to the AT(N) Research Framework of Alzheimer’s Disease

**DOI:** 10.3389/fnhum.2022.799347

**Published:** 2022-02-25

**Authors:** Miguel Ángel Rivas-Fernández, Mónica Lindín, Montserrat Zurrón, Fernando Díaz, José Manuel Aldrey-Vázquez, Juan Manuel Pías-Peleteiro, Laura Vázquez-Vázquez, Arturo Xosé Pereiro, Cristina Lojo-Seoane, Ana Nieto-Vieites, Santiago Galdo-Álvarez

**Affiliations:** ^1^Department of Clinical Psychology and Psychobiology, Universidade de Santiago de Compostela, Santiago de Compostela, Spain; ^2^Neurology Service, Santiago Clinic Hospital (CHUS), Santiago de Compostela, Spain; ^3^NeuroAging Group (NEURAL), Clinical Neurosciences Research Laboratory (LINC), Health Research Institute of Santiago de Compostela (IDIS), Santiago de Compostela, Spain; ^4^Department of Developmental and Educational Psychology, Universidade de Santiago de Compostela, Santiago de Compostela, Spain

**Keywords:** Alzheimer’s Disease (AD), AT(N), amyloid, tau, structural magnetic resonance imaging (sMRI), clinical diagnosis

## Abstract

**Introduction:**

This study aimed to evaluate, in adults with mild cognitive impairment (MCI), the brain atrophy that may distinguish between three AT(N) biomarker-based profiles, and to determine its clinical value.

**Methods:**

Structural MRI (sMRI) was employed to evaluate the volume and cortical thickness differences in MCI patients with different AT(N) profiles, namely, A−T−(N)−: *normal AD biomarkers*; A+T−(N)−: *AD pathologic change*; and A+T+(N)+: *prodromal AD*. Sensitivity and specificity of these changes were also estimated.

**Results:**

An initial atrophy in medial temporal lobe (MTL) areas was found in the A+T−(N)− and A+T+(N)+ groups, spreading toward the parietal and frontal regions in A+T+(N)+ patients. These structural changes allowed distinguishing AT(N) profiles within the AD continuum; however, the profiles and their pattern of neurodegeneration were unsuccessful to determine the current clinical status.

**Conclusion:**

sMRI is useful in the determination of the specific brain structural changes of AT(N) profiles along the AD continuum, allowing differentiation between MCI adults with or without pathological AD biomarkers.

## Introduction

Recently, the National Institute on Aging–Alzheimer’s Association (NIA-AA) proposed the AT(N) research framework in which Alzheimer’s disease (AD) is defined as a biological entity based on a dichotomous classification of normal (−) or abnormal (+) β-amyloid deposition (A), pathological tau (T), and neurodegeneration (N) biomarkers in living persons. While A and T are considered specific of AD, neuropathological (N) biomarkers [e.g., cerebrospinal fluid (CSF) total tau, FDG PET hypometabolism, and atrophy on MRI] are considered non-specific indicators of damage for AD which may derive from a variety of etiologies (Jack et al., 2016, 2018).

However, structural magnetic resonance imaging (sMRI) studies showed that atrophy progression in the AD continuum follows the stereotypical pattern of cortical tau tangles spread formalized into the Braak’s staging model (Braak and Braak, 1991). According to this model, cortical tau tangles are first observed in medial temporal lobe (MTL) structures, including the entorhinal cortex and hippocampus, then advances toward other MTL and limbic structures, and finally spread to frontal, parietal, and temporal cortical regions, as well as to subcortical structures, although different trajectories have been recently identified (Vogel et al., 2021). Therefore, the structural changes of brain assessed with MRI measures and progression could be used as objective and more specific indexes of neurodegeneration in the AD continuum (Bayram et al., 2018; Chandra et al., 2019).

Efforts have been made in finding more accurate neurodegenerative indices with precise anatomical location of gray matter (GM) or white matter (WM) changes. Allison et al. (2019) found that different MRI indices differentiated individuals across the AD continuum (Allison et al., 2019), with global atrophy being a robust general marker for neurodegeneration, equivalent to total tau measures, but, therefore, non-specific. Ekman et al. (2018) established a sequence of brain atrophy from A+T−(N)− to A+T+(N)− to A+T+(N)+; however, they measured the brain atrophy patterns using visual rating scales that only allow a global examination of neurodegenerative changes. Schwarz et al. (2016) recommended an AD signature combining several cortical thickness measures to distinguish the severity of AD (Schwarz et al., 2016), correlating with Braak’s neurofibrillary tangle staging, but this study did not distinguish between groups following the AT(N) framework.

Consequently, there is still a scarce evidence of the differences of brain atrophy between profiles proposed within the AT(N) framework. In addition, concerns about the prognostic value and clinical relevance of the profiles have also been raised (Morris et al., 2018). In this regard, it should be mentioned two recent studies conducted in elderly non-demented population (Ingala et al., 2021) and in adults with mild cognitive impairment (MCI), defined as a clinical syndrome in which activities of daily living are preserved but there is an objective cognitive impairment (Petersen et al., 2014), and patients with dementia of Alzheimer’s type (DAT) (Allegri et al., 2020).

Ingala et al. (2021) found that healthy non-demented population with positivity in phosphorylated tau (T +) showed cognitive dysfunction, especially in the memory domain, and non-demented adults with positivity in one or more AT(N) biomarkers (i.e., A+T−(N)−, A+T+(N)−, A+T−(N)+, and A+T+(N)+) displayed reduced volume in the amygdala, entorhinal cortex, and nucleus accumbens, showing an increase posterior cortical atrophy and cerebrovascular burden across the AD continuum profiles. Comparing cognitively unimpaired (CU) adults, individuals with early MCI or late MCI, and DAT patients, Allegri et al. (2020) found higher neurodegeneration in DAT patients compared with early MCI and late MCI subtypes and higher neurodegeneration in all patient groups (i.e., early MCI, late MCI, and DAT) compared with CU adults. In addition, they found that conversion to AD dementia was observed in a 60-month follow-up in 85% of individuals with the A+T+(N)+ profile.

Both investigations revealed important findings about the impact of AT(N) biomarker positivity on brain atrophy and cognitive function in non-demented adults, people with MCI, or patients with AD dementia. However, there is still a need for a more complete characterization of those neurodegenerative changes that occur in people with MCI given the AT(N) biomarker levels, especially in hippocampal subfields and also in other regions with an important relevance in AD dementia etiology, such as those areas included in the AD signature proposed by Schwarz et al. (2016), and its relationship with cognitive performance.

Consequently, and with the purpose of shedding more light on this unexplored area, the aims of this study were, first, to assess, using sMRI procedures, the brain structural changes, including a previously described index (i.e., AD signature index), that may distinguish between three AT(N) biomarker profiles [1], namely, (1) normal CSF biomarkers with MCI (A−T−(N)−), (2) AD pathologic change with MCI (A+T−(N)−), and (3) AD with MCI or prodromal AD (A+T+(N)+), and, second, to determine the clinical value of these profiles, i.e., to explore the relationship of subgroups of the AD continuum and the diagnosis of MCI subtypes and compare the cognitive performance between the three AT(N) biomarker profiles, using for that purpose, an extensive neuropsychological assessment.

Adults diagnosed with MCI were recruited, and CSF biomarkers were measured. Using sMRI, we aimed to evaluate between-group differences in GM and WM volume and cortical thickness at the whole brain with a follow-up region of interest (ROI) analysis in the hippocampal subfields and its surrounding MTL structures. We hypothesize that, compared with the other groups, adults with the A+T+(N)+ profile would display higher atrophy in the MTL and hippocampal subfields as well as in the frontal and posterior parietal cortices, and, therefore, atrophy in the AD signature index. We also expect worse performance in neuropsychological tests in the A+T+(N)+ group compared with the other groups.

## Materials and Methods

### Participants

A total of 37 adults with clinical diagnosis of MCI according to the standard criteria (Winblad et al., 2004; Albert et al., 2011) were selected from the longitudinal Compostela Aging Study and divided into three groups matched by age, gender, and years of education (refer to Table 1) as follows: 15 A−T−(N)− *normal AD biomarkers* (mean age: 69.67 years, SD: 5.41), 10 A+T−(N)− *AD pathologic change* (mean age: 72.60 years, SD: 9.98), and 12 A+T+(N)+ *prodromal AD* (mean age: 74.33 years, SD: 4.48).

**TABLE 1:** Mean values and standard deviations (SD, in brackets) of demographic, CSF, and neuropsychological measures.

	AT(N) biomarker profiles with MCI subtypes					

	A−T−(N)− *N* = 15 Mda-MCI *n* = 8 Sda-MCI *n* = 6 Mdna-MCI *n* = 1	A+T−(N)− *N* = 10 Mda-MCI *n* = 6 Sda-MCI *n* = 3 Mdna-MCI *n* = 1	A + T + (N)+ *N* = 12 Mda-MCI *n* = 10 Sda-MCI *n* = 2 Mdna-MCI *n* = 0	*p* *	Effect size Eta square (η^2^)	*Post hoc* comparisons
Age	69.67 (5.41)	72.60 (9.98)	74.33 (4.48)	0.202	0.057	NS^abcdef^
Years of education	7.27 (2.89)	9.20 (3.82)	6.25 (2.26)	0.082	0.113	NS
Gender (Female/Male)	9/6	6/4	9/3			
**CSF biomarkers**						
Amyloid (Aβ_42_)	1796.87 (558.40)	625.40 (163.52)	818.75 (167.70)	<0.001	0.663	<0.001^abc^
Phosphorylated tau (P-Tau)	40.30 (11.54)	50.32 (11.91)	116.79 (34.71)	<0.001	0.750	<0.001^ef^
Total tau (T-Tau)	272.47 (76.60)	310.70 (66.78)	711.08 (232.23)	<0.001	0.690	<0.001^ef^
Tau/Aβ	0.15 (0.04)	0.53 (0.22)	0.89 (0.31)	<0.001	0.734	<0.001^e^ /0.001^f^
**General cognitive functioning**						
MMSE	27.53 (1.46)	26.90 (2.69)	26.08 (2.23)	0.218	0.060	NS
CAMCOG-R (total)	87.07 (6.56)	82.50 (10.41)	77.50 (10.14)	0.032	0.124	0.027^b^
Subjective cognitive complains (patient)	14.80 (3.23)	17.00 (5.01)	14.75 (3.77)	0.326	0.017	NS
Subjective cognitive complains (informant)	15.46 (3.70)	16.60 (5.29)	18.90 (4.36)	0.155	0.170	NS
**Attention**						
TMT-A (seconds)	59.93 (25.85)	85.20 (52.15)	86.75 (30.76)	0.111	0.086	NS
CAMCOG-R (attention and calculation)	7.40 (1.45)	6.60 (2.37)	6.83 (1.19)	0.471	0.018	NS
**Executive function**						
TMT-B (seconds)	173.73 (67.13)	263.00 (217.31)	347.64 (162.76)	0.021	0.205	0.02^e^**
Phonological verbal fluency	11.20 (3.63)	9.50 (5.28)	9.50 (3.29)	0.462	0.052	NS
CAMCOG-R (executive function)	17.07 (3.86)	15.50 (5.46)	14.92 (3.63)	0.410	0.025	NS
**Memory**						
CVLT (short delay free recall)	5.93 (2.99)	5.70 (3.80)	5.75 (3.42)	0.983	0.00004	NS
CVLT (short delay cued recall)	8.27 (3.86)	7.10 (3.70)	6.50 (3.37)	0.452	0.033	NS
CVLT (long-delay free recall)	6.33 (4.25)	5.60 (4.12)	5.00 (3.79)	0.699	0.008	NS
CVLT (long-delay cued recall)	7.80 (3.90)	7.00 (3.40)	5.92 (3.20)	0.402	0.045	NS
CVLT (recognition hits)	14.40 (1.06)	14.30 (2.91)	13.83 (1.80)	0.737	0.149	NS
CVLT (recognition false positives)	3.53 (2.93)	5.40 (6.13)	8.42 (5.02)	0.035	0.129	0.03^e^
CAMCOG-R (memory)	20.27 (2.96)	18.50 (3.72)	15.25 (4.16)	0.004	0.216	0.003^b^
**Language**						
BNT	43.67 (4.62)	41.20 (11.15)	37.58 (10.15)	0.208	0.039	NS
Semantic verbal fluency (animals)	13.93 (2.87)	15.80 (7.90)	14.17 (2.73)	0.601	0.083	NS
CAMCOG-R (language)	25.07 (1.67)	25.40 (2.63)	24.33 (2.39)	0.503	0.034	NS
WAIS-R (vocabulary)	33.13 (11.18)	31.70 (18.14)	24.92 (11.66)	0.277	0.051	NS
**Functionality**						
Lawton and brody scale	7.40 (0.91)	7.00 (1.05)	7.33 (0.99)	0.586	0.048	NS
**Depression**					0.089	
Geriatric depression scale-15	2.40 (2.16)	4.10 (2.02)	2.83 (1.52)	0.109		NS

Sda-MCI, single-domain amnestic mild cognitive impairment, Mda-MCI, multiple-domain amnestic mild cognitive impairment; Mdna-MCI, multiple non-amnestic mild cognitive impairment. Post hoc comparisons: a, A−T−(N)− >A+ T−(N)−; b, A−T−(N)− > A+T+(N)+; c, A+T−(N)− > A+T+(N)+; d, A−T−(N)− < A+T−(N)−; e, A−T−(N)− < A+T+(N)+; f, A+T−(N)− < A+T+(N)+. *ANOVA (Group); MMSE, Mini-Mental State Examination; CVLT, California Verbal Learning Test, CAMCOG-R, Cambridge Cognitive Examination; BNT, Boston Naming Test; TMT, Trail Making Test; WAIS-R, Wechsler Adult Intelligence Scale – Revised. ** Two participants did not complete the TMT-B test, degrees of freedom were 34.

Participants gave their written informed consent prior the participation and did not report any previous diagnosis of any neurological disorder or psychiatric disturbances, history of clinical stroke, motor-sensory deficit, or substance abuse/dependence. All participants had normal audition and normal or corrected-to-normal vision and were right-handed as evaluated by the Edinburgh Handedness Inventory (Oldfield, 1971). The research project was approved by the Galician Clinical Research Ethics Committee and is in accordance with the 1964 Declaration of Helsinki ethical standards (Lynöe et al., 1991).

### Neuropsychological Assessment

Participants underwent extensive neuropsychological and cognitive assessment including the following tests: (a) short Spanish version of the “Questionnaire d’auto-évaluation de la Mémoire” (QAM) (Van der Linden et al., 1989) to evaluate subjective cognitive complains (SCCs) from patients and informants; (b) Mini-Mental State Examination (MMSE) (Folstein et al., 1975) and Cambridge Cognitive Examination – Revised (CAMCOG-R) (Roth et al., 2015) to evaluate general cognitive functioning and several cognitive domains, respectively; (c) California Verbal Learning Test (CVLT) (Delis et al., 1987) to assess memory; (d) Trail Making Test (TMT-A and TMT-B) (Reynolds, 2002) to evaluate attention and executive functioning; and (e) semantic (animals) and phonological (say in 1 min words starting with “p”) verbal fluency (Lezak et al., 2004), Boston Naming Test (BNT) (Kaplan et al., 1983), and the vocabulary test (Wechsler Adult Intelligence Scale – Revised or WAIS-R) (Weschler, 2002) to assess language. Moreover, functional assessment was done using the Lawton and Brody Index (maximum possible scoring = 8) to evaluate instrumental activities of daily living (IADL) (Lawton and Brody, 1969) and depression was evaluated with the Geriatric Depression Scale (GDS-15) (Sheikh and Yesavage, 1986).

Mild cognitive impairment was diagnosed following the criteria proposed by Albert et al. (2011) as follows: (i) evidence of concern about a change in cognitive function, in comparison with the previous level corroborated by informants (QAM); (ii) evidence of poorer performance in one or more cognitive domains that is greater than expected for the age and educational background of patient; for this criterion, we established to have a performance score of 1.5 SD below age- and education-related norms in general cognitive functioning (MMSE) and in one or more cognitive domains (CAMCOG-R and CVLT); (iii) preservation of independence (IADL); and (iv) non-fulfillment of diagnostic of dementia according the *DSM-V* (*Diagnostic and Statistical Manual of Mental Disorders, 5th Edition*) and NIA-AA criteria. Besides, participants were classified following the criteria proposed by Petersen (2004) and Winblad et al. (2004) into three types: (i) individuals with multiple-domain amnestic MCI (mda-MCI) (*n* = 24) who scored 1.5 SD below age- and education-related norms in the MMSE, and in at least two CAMCOG-R subscales and also in the CVLT (short and long delay free recall); (ii) individuals with single-domain amnestic MCI (sda-MCI) (*n* = 11), with normal cognitive functioning in the MMSE and CAMCOG-R subscales, but with memory impairment in the CVLT; and (iii) individuals with multiple-domain non-amnestic MCI (mdna-MCI) (*n* = 2) with normal memory functioning, but with cognitive impairments as in the mda-MCI type. Diagnoses were reached by consensus within the research team.

### AT(N) Biomarker Measurement

Participants underwent a lumbar puncture, following the protocolized recommendations to standardize pre-analytical confounding factors in AD CSF biomarkers (Del Campo et al., 2012). CSF biomarkers [e.g., β-amyloid(1–42), hTAU-Ag, and phospo-tau 181P] were assessed with INNOTEST^®^ sandwich ELISA (Fujirebio Europe, Ghent, Belgium) according to the procedures of the manufacturer. All the data were generated in a single center through the routine activity of the Clinical Neuroscience Research Laboratory (LINC) at the Health Institute of Santiago de Compostela (IDIS) as described by Jongbloed et al. (2013). Due to the number of included patients, the lots of assay kits were variable. However, the quality of the results was ensured by the use of validated standard operating procedures and internal quality controls (QCs). The range of the QC coefficient of variation for Aβ42 across the different lots was 12–13%. For tau and p-tau, the range was 10–13% and 7.3–11%, respectively. The range of the QC intra-assay coefficient of variation was <8% for all biomarkers.

The relationship of Aβ1-42, t-Tau, 181p-Tau, and ratios of their cutoff levels with test result was measured with INNOTEST^®^ as follows: if Aβ1-42 < 638 pg/ml, the test result is positive; if t-Tau >375 pg/ml, the test result is positive; if 181p-Tau >52 pg/ml, the test result is positive; and if t-Tau/Aβ1–42 ratio >0.52, the test result is positive. These cutoff points were calculated by Fujirebio specifically for the LINC following the Alzheimer’s Biomarkers Standardization Initiative (Molinuevo et al., 2014).

### Magnetic Resonance Imaging Acquisition and Data Analysis

For sMRI analysis, a sagittal T1-weighted 3D-MPRAGE sequence (i.e., repetition time/echo time = 7.45 ms/3.40 ms, flip angle = 8°; 180 slices, voxel size = 1 × 1 × 1 mm, field of view = 240 × 240 mm^2^, and matrix size = 240 × 240 mm) was acquired on a Philips 3T Achieva scanner (Philips Medical System, Best, Netherlands) at the University Hospital Complex in Santiago de Compostela, Galicia (Spain). Head motion was minimized by using a head restraint system and by placing foam padding around the head of subjects. Participants were provided with headphones to attenuate scanner noise.

The differences in GM and WM volume were evaluated performing a voxel-based morphometry analysis in Matlab R2016a using the Computational Anatomy Toolbox^1^ implemented in the Statistical Parametric Mapping software (SPM12).^2^ T1-weighted images were visually inspected and reoriented to the anterior-posterior commissure, segmented in GM and WM tissues (Ashburner and Friston, 2005), and normalized to the Montreal Neurological Institute (MNI) space using a customized template built with the DARTEL toolbox (Ashburner, 2007). Next, the normalized GM/WM-modulated images were smoothed using a Gaussian kernel Full Width at Half Maximum (8-mm FWHM). Statistical analyses were conducted using the generalized linear modeling (GLM) approach and between-group analysis was performed using the normalized and smoothed GM- and WM-modulated images *via* 2 one-way analysis of variance (ANOVA) tests with a between-subject factor *Group* (three levels: A−T−(N)−, A+T−(N)−, and A+T+(N)+) including the total intracranial volume as covariate. Results were assessed at *p* < 0.05 with family-wise error (FWE) cluster-level corrected for multiple comparisons combined with a threshold of *p* < 0.001 at the uncorrected voxel level.

Cortical thickness differences were evaluated performing a surface-based morphometry analysis using the FreeSurfer version 6.0 software.^3^ We employed the automated default preprocessing pipeline of cortical reconstruction and volumetric segmentation (Dale et al., 1999) that included motion correction (Reuter et al., 2010), skull stripping (Segonne et al., 2004), transformation into the Talairach space, segmentation of cortical and subcortical GM/WM volumetric structures (Fischl et al., 2002, 2004), intensity normalization (Sled et al., 1998), tessellation of the boundary between GM and WM, and topology correction (Segonne et al., 2007). Pial and WM segmentations were visually inspected and corrected when necessary. Between-group analysis was performed *via* a GLM with a Monte Carlo simulation multiple comparisons correction with 10,000 permutations, a cluster-forming threshold set at *p* < 0.005 and a smoothing kernel of 15-mm FWHM. Results were considered significant at *p* < 0.05.

**FIGURE 1:**
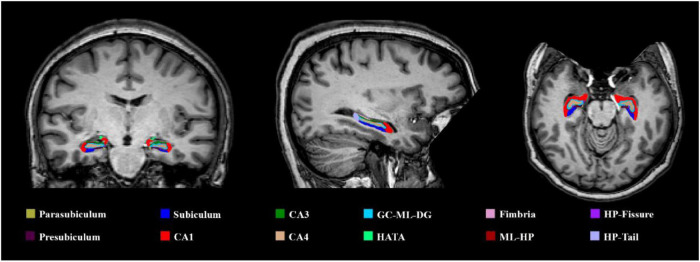
An illustrative example of the hippocampal subfield segmentation performed in FreeSurfer. CA, cornu ammonis; GC-ML-DG, granule cell of the molecular layer of dentate gyrus; ML-HP, molecular layer of the hippocampus; HATA, hippocampus-amygdala-transition-area; HP-fissure, hippocampus fissure; HP-tail, hippocampus tail.

A follow-up ROI analysis was performed over MTL given that the whole-brain analysis revealed significant differences in this area (refer to Table 2). Hippocampal subfields were automatically segmented with FreeSurfer (Iglesias et al., 2015) (refer to Figure 1). After a visual QC, these volume measurements were exported: whole hippocampus and its head, body, and tail; parasubiculum; and head and body of presubiculum, subiculum, CA1, CA3 (CA2 is included in CA3), CA4, granulate cell of the molecular layer of dentate gyrus, hippocampal molecular layer, hippocampal fissure, fimbria, and the hippocampus-amygdala transition area. The entorhinal cortex and parahippocampal gyrus volume and all hippocampal subfields volume measurements were adjusted using the estimated total intracranial volume (eTIV) employing a formula: adjusted_volume = volume_observed - *b* × (eTIV - mean_eTIV), where mean_eTIV is the average eTIV of all subjects and *b* is the regression coefficient between the volume observed and the eTIV. In comparison to other adjustment approaches, this adjustment method, also known as residual approach, demonstrated to be optimal for discriminating not only between CU adults and individuals with AD dementia but also between people with MCI and adults with AD dementia (Voevodskaya, 2014). Between-group analysis was performed using a multivariate GLM including the Group as the fixed factor and all the volume measures as dependent variables. The Holm-Bonferroni method was employed to correct for multiple comparisons, and significance level was set at *p* < 0.05.

**TABLE 2:** Brain regions that showed significant gray matter (GM) and white matter (WM) volume differences and cortical thickness differences in the between-group analyses.

	Brain region	Cluster size	L/R	MNI Coordinates			Statistic		Cohen’s D	
				X	Y	Z	F	*t*		
**Volume differences**	**Gray matter**									
	NS									
	**White matter**									
	**Group effect**									
	Parahippocampal gyrus	9035	L	−30	−20	−22	17.17			
	Hippocampus		L	−30	−22	−16	16.82			
	Middle temporal gyrus		L	−47	−11	−18	12.65			
	Inferior temporal gyrus		L	−46	−34	−19	11.75			
	**A−T−(N)− > A+T−(N)−**									
	Parahippocampal gyrus	2927	L	−31	−17	−23		4.42	1.54	
	Hippocampus		L	−29	−23	−15		3.91	1.36	
	**A−T−(N)− > A+T+(N)+**									
	Parahippocampal gyrus	16339	L	−30	−21	−21		5.55	1.93	
	Hippocampus		L	−30	−22	−16		5.54	1.93	
	Lingual gyrus		L	−19	−40	0		5.21	1.81	
	Middle temporal gyrus		L	−47	−11	−18		5.01	1.75	
	Inferior temporal gyrus		L	−46	−29	−18		4.26	1.48	
	Inferior temporal gyrus	3031	R	51	−15	−22		5.13	1.79	
	Fusiform gyrus		R	41	−12	−29		3.81	1.34	
	Middle frontal gyrus (orbital part)	2885	R	29	44	−8		4.39	1.53	
	Anterior cingulate		R	9	34	−8		3.98	1.39	

	**Brain region**	**Cluster size** **(mm^2^)**	**L/R**	**MNI coordinates**			**Max** **−log10(*p*-value)**		**CWp**	**Cohen’s D**
				**X**	**Y**	**Z**				

**Cortical thickness**	**Group effect**									
	Lateral occipital cortex	907.39	L	−21.8	−95.9	1.9	4.40		0.001	
	Lateral occipital cortex	1057.15	R	29.3	−88.6	1.8	5.46		0.0006	
	Inferior parietal lobule	655.76	L	−31.5	−70.5	37.5	5.40		0.017	
	**A−T−(N)− > A+T+(N)+**									
	Inferior parietal lobule	3917.90	L	−31.5	−70.5	37.5	6.07		0.0002	1.33
	Precuneus	1501.33	L	−9.3	−62.8	50.6	4.29		0.0002	1.32
	Posterior cingulate	1363.30	L	−13.5	−34.5	38.8	3.40		0.0002	1.21
	Entorhinal cortex	838.52	L	−31.5	−13.2	−30.7	2.69		0.0117	1.14
	Supramarginal gyrus	808.76	L	−54.7	−50.5	15.6	3.68		0.0144	1.20
	Lateral occipital cortex	1605.22	R	29.3	−88.6	11.8	6.11		0.0004	1.53
	Superior frontal gyrus	619.87	R	11.0	11.4	62.8	5.11		0.0394	1.47

*L/R, left or right hemisphere; Max -log10(p-value), Maximum -log10(p-value) at each cluster; MNI, montreal neurological institute coordinates; CWP, cluster-wise p-value; Cohen’s D, effect sizes; NS, not significant.*

To contrast the relationship between groups (established by CSF AT(N) profiles) and clinical diagnosis (different MCI subtypes), a chi-squared test was carried out. One-way ANOVAs with CSF biomarker profile as main factor with three levels, namely, A−T−(N)−, A+T−(N)−, and A+T+(N)+, were performed to compare the cognitive performance in a battery of neuropsychological tests, and pairwise comparisons corrected to Holm–Bonferroni were performed in case of significant effects (*p* < 0.05). Moreover, AD signature (Schwarz et al., 2016) was computed by averaging the FreeSurfer thickness estimates from the entorhinal cortex, inferior temporal gyrus, middle temporal gyrus, inferior parietal lobe, fusiform gyrus, and precuneus. To determine the sensitivity and specificity of the brain structural changes to distinguish between AT(N) profiles, receiver operating characteristic (ROC) curve analyses were performed.

## Results

### Structural Magnetic Resonance Imaging

**FIGURE 2:**
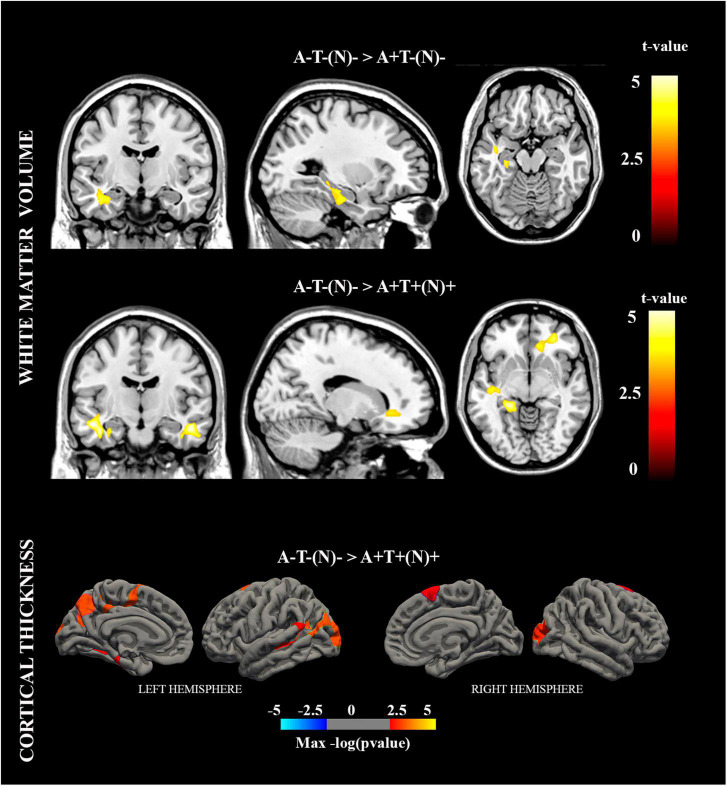
Brain regions with significant white matter (WM) volume and cortical thickness differences in the between-group analyses.

The WM volume showed a significant group effect in the parahippocampal gyrus, hippocampus, and inferior/middle temporal gyrus of the left hemisphere. Compared with the A−T−(N)− group, the A+T−(N)− group showed a significant reduced WM volume in the parahippocampal gyrus and hippocampus of the left hemisphere, and the A+T+(N)+ group showed a significant reduced WM volume in the parahippocampal gyrus, hippocampus, lingual gyrus, and middle temporal gyrus of the left hemisphere; bilateral inferior temporal gyrus; and fusiform gyrus, middle frontal gyrus (orbital part), and anterior cingulate of the right hemisphere (refer to Figure 2 and Table 2). No significant group effect was found in the GM volume.

Cortical thickness showed a significant group effect in the left inferior parietal lobule and bilateral lateral occipital cortex. Compared with the A−T−(N)− group, the A+T+(N)+ group showed a significant thinning in the inferior parietal lobule, precuneus, posterior cingulate, supramarginal gyrus, and entorhinal cortex of the left hemisphere and in the lateral occipital cortex and superior frontal gyrus of the right hemisphere (refer to Figure 2 and Table 2).

The follow-up ROI analyses of MTL (refer to Table 3) showed, for brain volume and thickness, significant group effects. A+T+(N)+ participants showed reduced volume compared with A−T−(N)− participants at the bilateral regions (e.g., whole hippocampus and its head, body, and tail; head and body of subiculum; head of CA1; head and body of the hippocampal molecular layer; and the entorhinal cortex) and left regions (e.g., head and body of presubiculum, body of CA1, body of CA3, head and body of CA4, head and body of granulate cell of dentate gyrus, and parahippocampal gyrus). Also, a cortical thickness reduction in the bilateral entorhinal cortex and parahippocampal gyrus was found.

**TABLE 3:** Mean values and standard deviations (SD, in brackets) of the adjusted hippocampal subfields measures and its surrounding medial temporal lobe regions in which the between-group analyses revealed significant volume and thickness differences.

Brain region			A−T−(N)− *N* = 15		A+T−(N)− *N* = 10		A+T+(N)+ *N* = 12		*p*		Effect size η^2^		*Post hoc* comparisons	
							
			L	R	L	R	L	R	L	R	L	R	L	R
**Volume (mm^3^)**														
Hippocampus	Whole hippocampus		3162.22 (270.60)	3285.55 (295.83)	2981.67 (442.52)	3077.44 (403.12)	2587.07 (314.99)	2802.40 (347.79)	**<0.001**	**0.004**	0.37	0.28	<0.001^b^/0.03^c^	0.003^b^
	Hippocampus	Head	1552.85 (115.53)	1646.88 (140.52)	1477.61 (291.48)	1544.09 (247.27)	1293.41 (175.80)	1414.70 (191.45)	**0.006**	**0.013**	0.26	0.23	0.005^b^	0.01^b^
		Body	1094.07 (106.90)	1103.75 (110.93)	1019.60 (120.88)	1042.70 (139.34)	886.86 (101.75)	951.34 (135.34)	**<0.001**	**0.014**	0.42	0.22	<0.001^b^/0.02^c^	0.01^b^
		Tail	515.29 (79.74)	534.93 (67.22)	484.46 (62.93)	490.66 (52.47)	406.80 (68.14)	436.36 (55.17)	**0.002**	**0.001**	0.32	0.35	0.001^b^/0.05^c^	<0.001^b^
	Presubiculum	Head	129.14 (9.43)	129.05 (15.12)	122.21 (23.30)	120.17 (21.91)	108.16 (16.73)	114.61 (17.93)	**0.009**	0.126	0.24	0.12	0.007^b^	NS
		Body	145.90 (16.14)	130.21 (18.66)	136.22 (22.26)	127.07 (21.52)	125.46 (20.74)	118.34 (14.75)	**0.035**	0.249	0.18	0.09	0.03^b^	NS
	Subiculum	Head	179.28 (17.74)	180.53 (24.42)	166.90 (34.68)	165.95 (25.34)	143.48 (21.01)	151.98 (22.15)	**0.002**	**0.015**	0.30	0.22	0.002^b^	0.01^b^
		Body	233.10 (23.47)	224.95 (25.70)	211.97 (24.91)	210.28 (23.66)	191.16 (28.55)	195.56 (31.41)	**0.001**	**0.030**	0.35	0.19	<0.001^b^	0.03^b^
	Parasubiculum		62.34 (13.22)	62.76 (14.42)	60.21 (14.45)	62.44 (18.95)	55.62 (13.79)	54.39 (13.66)	0.453	0.331	0.05	0.06	NS	NS
	CA1	Head	469.25 (48.52)	506.35 (47.78)	444.25 (91.84)	474.19 (82.92)	391.37 (55.48)	430.42 (47.47)	**0.014**	**0.008**	0.22	0.25	0.01^b^	0.006^b^
		Body	112.16 (17.85)	118.49 (11.95)	110.46 (21.39)	121.49 (19.17)	89.20 (17.45)	103.98 (15.36)	**0.007**	**0.020**	0.25	0.21	0.01^b^/0.04^c^	0.03^c^
	CA3*	Head	108.27 (17.10)	119.96 (13.57)	108.43 (24.85)	114.22 (24.50)	91.48 (19.45)	107.36 (29.71)	0.073	0.373	0.14	0.06	NS	NS
		Body	83.62 (15.16)	96.21 (14.20)	83.55 (18.27)	93.17 (17.27)	65.61 (11.29)	81.41 (16.00)	**0.007**	0.055	0.26	0.16	0.01^b^/0.03^c^	NS
	CA4	Head	113.97 (9.64)	123.29 (11.11)	111.41 (22.13)	118.14 (21.66)	96.31 (15.75)	107.33 (22.12)	**0.017**	0.090	0.21	0.13	0.02^b^	NS
		Body	113.20 (14.77)	118.09 (16.65)	107.46 (14.43)	116.16 (18.00)	91.75 (10.44)	103.33 (18.62)	**0.001**	0.091	0.34	0.13	0.001^b^/0.03^c^	NS
	GC.ML.DG	Head	136.49 (11.78)	148.51 (13.89)	131.11 (28.46)	139.66 (28.12)	113.93 (18.99)	128.28 (26.47)	**0.017**	0.085	0.21	0.14	0.02^b^	NS
		Body	126.49 (15.91)	130.84 (16.70)	117.90 (14.65)	126.51 (20.07)	102.93 (11.93)	113.11 (20.32)	**0.001**	0.059	0.35	0.15	0.001^b^	NS
	Molecular Layer HP	Head	303.01 (23.32)	318.38 (28.69)	284.30 (58.30)	296.98 (50.53)	247.36 (36.08)	271.12 (34.52)	**0.003**	**0.01**	0.29	0.24	0.003^b^	0.008^b^
		Body	206.70 (23.60)	212.08 (22.59)	193.07 (26.65)	200.28 (28.54)	162.60 (22.36)	178.48 (26.07)	**<0.001**	**0.006**	0.40	0.26	<0.001^b^/0.02^c^	0.005^b^
	Hippocampal fissure		163.18 (14.78)	185.85 (25.81)	156.94 (21.12)	185.17 (34.30)	147.56 (26.73)	171.32 (31.60)	0.172	0.413	0.1	0.05	NS	NS
	Fimbria		72.90 (16.42)	72.88 (16.39)	59.01 (19.22)	47.73 (17.05)	58.13 (17.47)	57.15 (15.92)	0.063	**0.002**	0.15	0.31	NS	0.002^a^
	HATA		51.10 (11.01)	58.05 (10.01)	48.81 (9.54)	52.35 (7.74)	45.70 (10.27)	49.19 (10.73)	0.416	0.069	0.05	0.15	NS	NS
PHG			2104.27 (401.77)	1796.87 (188.54)	1675.44 (295.41)	1634.61 (311.44)	1741.55 (255.46)	1592.15 (274.16)	**0.005**	0.102	0.27	0.13	0.010^a^/0.024^b^	NS
Entorhinal C.			1632.67 (237.70)	1727.19 (257.15)	1345.71 (369.77)	1445.52 (438.87)	1205.49 (278.36)	1374.83 (334.94)	**0.002**	**0.025**	0.31	0.20	0.002^b^	0.03^b^
**Thickness(mm)**														
PHG			2.66 (0.28)	2.54 (0.21)	2.44 (0.30)	2.34 (0.21)	2.39 (0.22)	2.31 (0.26)	**0.025**	**0.025**	0.20	0.20	0.03^b^	0.04^b^
Entorhinal C.			3.22 (0.28)	3.27 (0.41)	2.85 (0.59)	2.85 (0.49)	2.73 (0.56)	2.83 (0.48)	**0.030**	**0.025**	0.19	0.20	0.04^b^	0.05^b^

L/R, left or right hemisphere; PHG, parahippocampal gyrus; Entorhinal C, entorhinal cortex; CA, cornu ammonis; GC.ML.DG, granule cell of the molecular layer of dentate gyrus; Molecular Layer HP, Molecular layer of the hippocampus; HATA, hippocampus-amygdala-transition-area; η^2^, effect size Eta square *, CA2 is included in the CA3 subfield. Post hoc comparisons: a, A−T−(N)− > A+T−(N)−; b, A−T−(N)− > A+T+(N)+; c, A+T−(N)− > A+T+(N)+. p values of those brain regions in which it was found a significant group effect are displayed in bold.

A+T+(N)+ participants showed reduced volume compared with A+T−(N)− participants at the bilateral regions (e.g., body of CA1) and left regions (e.g., whole hippocampus and its body and tail, body of CA3, body of CA4, and body of the hippocampal molecular layer).

A + T−(N)− participants showed reduced volume compared with A−T−(N)− participants at the right fimbria and left parahippocampal gyrus.

For the AD signature index, the ANOVA revealed a significant effect of the group factor, given that this index was larger for A+T+(N)+ participants than that for the A+T−(N)− (*p* = 0.048) and A−T−(N)− (*p* = 0.003) participants. Table 4 shows the ROC curve results. The AD signature index revealed two AUC values over 0.80, with high sensitivity and specificity to distinguish between A+T+(N)+ and A−T−(N)− and the profiles of AD continuum (A+T+(N)+ and A+T−(N)−) with respect to the A−T−(N)− profile.

**TABLE 4:** Receiver operating characteristic (ROC) curve results.

AT(N) Groups	Cut-off	AUC	Sensitivity	Specificity
AD signature				
A−T−(N)− and A+T−(N)−	2.5352	0.75 (0.54 to 0.97)	0.70 (0.35 to 0.93)	0.73 (0.45 to 0.92)
A−T−(N)− and A+T+(N)+	2.4931	0.89 (0.76 to 1.00)	1.00 (0.74 to 1)	0.80 (0.52 to 0.96)
A+T−(N)− and A+T+(N)+	2.4438	0.55 (0.28 to 0.82)	0.67 (0.35 to 0.90)	0.50 (0.19 to 0.81)
A−T−(N)− and [A+T−(N)− and A+T+(N)+]	2.5352	0.83 (0.69 to 0.97)	0.86 (0.65 to 0.97)	0.73 (0.45 to 0.92)
[A−T−(N)− and A+T−(N)−] and A+T+(N)+	2.4931	0.75 (0.60 to 0.91)	1.00 (0.74 to 1)	0.64 (0.43 to 0.82)

*AUC, area under the curve. Lower and upper limits of 95% confidence intervals in brackets.*

### Neuropsychological Assessment

Analysis of variances showed a significant group effect only in four different cognitive tests (refer to Table 1). Concretely, A+T+(N)+ presented worse performance than A−T−(N)− in the total score of CAMCOG-R, the memory test of CAMCOG-R, false positive in recognition of CVLT, and TMT-B. No further significant differences were found between groups. Moreover, chi-squared test revealed no differences in the proportion of the different MCI subtypes into the three profiles of the AT(N) classification (χ^2^ = 3.28; *p* = 0.512; refer to proportions in Table 1).

## Discussion

This study had two aims: to obtain the indices of brain atrophy that may distinguish between three profiles within the AT(N) framework and to determine the clinical value of this classification, thereby relating it to the cognitive performance and clinical status.

The results confirm that the profiles based on CSF biomarkers are useful to estimate the degree of brain atrophy. Consistent with the results reported by Ekman et al. (2018) using visual rating scales, a gradation in brain atrophy indices (WM volume and/or cortical thickness) is appreciated, which allows distinguishing not only between those participants with normal AD biomarker (A−T−(N)−) profile and the AT(N) profiles of the AD continuum but also between those with the AD pathologic change (A+T−(N)−) and the prodromal AD (A+T+(N)+) profiles.

Specifically, compared with A−T−(N)− participants, the A+T−(N)− profile displayed atrophy in the right fimbria and left parahippocampal gyrus, but the A+T+(N)+ group showed more severe degeneration in several MTL structures (e.g., parahippocampal gyrus, entorhinal cortex, and hippocampal subfields). The WM volume changes in the parahippocampal gyrus were previously reported in AD patients (Li et al., 2012) and represent a possible biomarker of disease progression (Solodkin et al., 2013). The entorhinal cortex shows the earliest neurodegenerative changes in AD (Leal and Yassa, 2013), and its thinning predicts hippocampal atrophy when abnormal amyloid and p-tau elevated levels are present in MCI and AD dementia (Desikan et al., 2010). Moreover, this region is an important hub of the perforant pathway, a tract essential for episodic memory function by which the entorhinal cortex communicates to the dentate gyrus and other hippocampal subfields the inputs received from multiple cortical areas (Witter, 2007). The fimbria establish connections with the fornix, forming another important tract that connects the hippocampus with the hypothalamus (Leal and Yassa, 2013). Recent research demonstrated that neural damage in the fornix is related to amyloid burden and episodic memory decline (Rabin et al., 2019). Although other neuroimaging modalities (e.g., diffusion tensor imaging) can assess in more detail the microstructural damage of these tracts, the volume changes found in the fimbria in the A+T−(N)− group, and the extensive damage displayed by the A+T+(N)+ group in the entorhinal cortex and several hippocampal subfields, suggest a progressive disintegration of two brain networks functionally related to episodic memory, the neurocognitive system most affected in AD dementia.

Moreover, in line with the progression of subsequent stages of development of AD (Braak and Braak, 1991), the A+T+(N)+ participants showed, compared with A−T−(N)− participants, WM reductions in the temporal (inferior/middle temporal gyrus), frontal (orbital part of middle frontal gyrus), and occipito-temporal (fusiform and lingual gyrus) areas and the anterior cingulate cortex. This group also displayed a significant thinning in cortical thickness of the inferior parietal lobule, precuneus, posterior cingulate, supramarginal gyrus, superior frontal gyrus, and the lateral occipital cortex. Overall, these structural changes found in MTL and occipito-temporal regions suggest that neurodegeneration is affecting regions with an important role in the consolidation and retrieval of episodic memories. However, the increased brain atrophy found in the A+T+(N)+ group seems to indicate that AD progression is expanding toward additional regions of parietal and frontal cortex involved in other cognitive domains, such as executive control.

These changes in brain integrity of the AD continuum profiles and the expected neurodegeneration process in AD according to Braak’s neurofibrillary tangle staging are especially evident in the structures that conform the AD signature index (Schwarz et al., 2016). In fact, ROC analyses evidenced that this index distinguished with high specificity and sensitivity between participants within the AD continuum and those with normal CSF levels of amyloid and tau. Given this clear relationship, it is possible that the AT(N) profiles represent a good predictor of disease progression, in line with previous reports (Bakkour et al., 2009; Dickerson and Wolk, 2013). Our results also indicate that exploring the AD signature may be useful as a biomarker to estimate that patients are in the AD continuum and to determine the current state of global neurodegeneration, regardless their cognitive performance and clinical status.

Precisely, once the validity of the CSF AT(N) framework in terms of brain atrophy has been confirmed, another aim of this study focused on checking the clinical relevance of these diagnostic profiles. In this sense, consistent with the brain atrophy results, the most extreme groups of the AT(N) framework (A−T−(N)− vs. A+T+(N)+) showed differences in a global measure of cognitive performance (CAMCOG-R Total), two measures of memory (CAMCOG-R Memory and CVLT false positives in recognition), and a measure of executive functions (TMT-B). However, no further differences were found between groups.

Previous investigations conducted in non-demented adults demonstrated that positivity in phosphorylated tau (T +) drives cognitive dysfunction, especially in the memory domain (Ingala et al., 2021), although there is also evidence that cognitive deficits in episodic memory, executive function, and global cognitive function are positively associated with amyloid burden in adults without a diagnosis of MCI or AD dementia (Hedden et al., 2013). Highly consistent with the present results, Ekman et al. (2018) found reduced episodic memory performance in MCI adults with an overall biomarker positivity (A+T+(N)+) in comparison to their counterparts with the A−T−(N)− profile. Thus, taking into account this previous evidence, the present results support the suggested idea that A+T+(N)+ profile represents an advanced-stage disease within the AD continuum in which disease progression is affecting the functioning of several cognitive domains, such as episodic memory or executive function (Allegri et al., 2020; Junquera et al., 2020).

Furthermore, no relationship was found between biomarker-based profiles in CSF and MCI clinical diagnosis. In fact, similar proportions of the different MCI subtypes were found in all three profiles. Therefore, AT(N) profiles do not seem to correspond with a progression in clinical severity among the different MCI subtypes, considering mda-MCI the most severe and sda-MCI the less severe (Brambati et al., 2009). Previous studies have already shown a lack of correspondence between CSF biomarker profiles and cognitive performance (Jack et al., 2019). Thus, as suggested in previous studies, there is a dissociation between the biomarker profile within the AD continuum and clinical status (Jack et al., 2019). Other factors such as the cognitive reserve may be involved in this dissociation (Facal et al., 2019; Stern et al., 2020).

The implications of these findings are present at different levels. In contrast, many studies have reported that the discrepancy in diagnostic criteria with respect to MCI may explain the divergence of results in previous literature in terms of characterization of brain integrity and activity in participants with MCI (Brambati et al., 2009), but, in line with the assessment of the NIA-AA (Jack et al., 2018), these discrepancies may be modulated by pathophysiological features in terms of the presence of amyloid and pathological tau. It is possible that, at least partially, this neuropathological heterogeneity of participants with MCI may also explain the variability in conversion rates to DAT found in previous studies (Facal et al., 2015). In contrast, even sharing a similar state of neurodegenerative changes, there does not seem to be a direct relationship between biomarker status and clinical status of a person. Therefore, apart from the importance of characterizing patients according to their biomarkers for the development of specific therapies that seek to slow the progression of neuropathological processes, the data obtained emphasize the need to obtain possible (e.g., genetic, molecular, and cognitive) triggering and/or protective factors that translate the neurodegenerative process associated with the AD continuum into the appearance of relevant clinical symptoms, with the aim of preventing and/or treating the appearance of symptoms that affect the cognitive performance of patients.

Finally, some limitations of this study are worth noting. Although no age differences were obtained between the three AT(N) groups, further research with larger samples is needed to explore the interaction between age and the CSF biomarker profiles. Also, a larger sample would allow to focus on possible interactions between AT(N) profiles and/or clinical diagnosis with different cognitive domains.

## Conclusion

In sum, the study of sMRI measures revealed a progressive neurodegeneration along the AT(N) profiles consistent with the change pattern in AD (initial damage of MTL areas spreading toward the posterior parietal and frontal regions). Furthermore, the AD signature index, measuring the cortical thickness in brain regions vulnerable to AD, presented moderate-to-high sensitivity and specificity distinguishing the profiles within the AD continuum compared with those with normal CSF biomarkers. However, the AT(N) profiles and their pattern of degeneration were unsuccessful to determine the current clinical status; further research should clarify the factors that prevent or facilitate poorer cognitive performance and the presence of clinical symptoms given a certain amount of brain atrophy.

## Data Availability Statement

The raw data supporting the conclusions of this article will be made available by the authors, without undue reservation.

## Ethics Statement

The studies involving human participants were reviewed and approved by Galician Clinical Research Ethics Committee. The patients/participants provided their written informed consent to participate in this study.

## Author Contributions

MR-F contributed to the design of methodology, conducted data collection and formal analyses, wrote the initial draft, and participated in the preparation of the published work. ML contributed to conceptualization and formulation of research goals, manuscript revision, design of methodology, data collection, preparation of the published work, and supervision. MZ and FD contributed to conceptualization and formulation of research goals, manuscript revision, data collection, provision of study materials, supervision, project administration, and funding acquisition. JA-V and JP-P contributed to conceptualization and formulation of research goals, design methodology, data collection, and formal analyses. LV-V contributed to CSF biomarker characterization. AP contributed to conceptualization and formulation of research goals, design methodology, data collection, formal analyses, and project administration. CL-S and AN-V contributed to data collection and formal analyses. SG- contributed to conceptualization and formulation of research goals, manuscript revision, design of methodology, data collection, preparation of the published work, and supervision.

## Conflict of Interest

The authors declare that the research was conducted in the absence of any commercial or financial relationships that could be construed as a potential conflict of interest.

## Publisher’s Note

All claims expressed in this article are solely those of the authors and do not necessarily represent those of their affiliated organizations, or those of the publisher, the editors and the reviewers. Any product that may be evaluated in this article, or claim that may be made by its manufacturer, is not guaranteed or endorsed by the publisher.

## Footnotes

1
http://www.neuro.uni-jena.de/cat/

2
https://www.fil.ion.ucl.ac.uk/spm/

3
http://surfer.nmr.mgh.harvard.edu/
